# Awake Craniotomy in Epilepsy Surgery: A Case Series and Proposal for Three Different Scenarios

**DOI:** 10.3390/brainsci14100958

**Published:** 2024-09-25

**Authors:** Takehiro Uda, Yuta Tanoue, Toshiyuki Kawashima, Vich Yindeedej, Shugo Nishijima, Noritsugu Kunihiro, Ryoko Umaba, Kotaro Ishimoto, Takeo Goto

**Affiliations:** 1 Department of Neurosurgery, Graduate School of Medicine, Osaka Metropolitan University, Osaka 545-8585, Japan; 2 Department of Pediatric Neurosurgery, Osaka City General Hospital, Osaka 534-0021, Japan; 3 Division of Neurosurgery, Department of Surgery, Thammasat University Hospital, Faculty of Medicine, Thammasat University, Pathumthani 12120, Thailand

**Keywords:** awake craniotomy, epilepsy surgery, indication, strategy, general anesthesia

## Abstract

Objective: Awake craniotomy (AWC) allows intraoperative evaluation of functions involving the cortical surface and subcortical fibers. In epilepsy surgery, indications for and the role of AWC have not been established because evaluation with intracranial electrodes is considered the gold standard. We report herein our case series of patients who underwent AWC in epilepsy surgery and propose the scenarios for and roles of AWC. Methods: Patients who underwent AWC in epilepsy surgery at our institutions between 2014 and 2023 were included. Information about age, sex, etiology, location of epileptogenicity, seizure type, use of intracranial electrode placement, surgical complications, neurological deficits, additional surgery, and seizure outcomes was reviewed. Following a diagnostic and treatment flow for epilepsy surgery, we clarified three different scenarios and roles for AWC. Results: Ten patients underwent AWC. Three patients underwent AWC after non-invasive evaluations. Two patients underwent AWC after intracranial evaluation with stereotactic electroencephalography (SEEG). Five patients underwent AWC after intracranial evaluation with subdural grid electrodes (SDG). Among these, two patients were initially evaluated with SEEG and with SDG thereafter. One patient reported slight numbness in the hand, and one patient showed slight cognitive decline. Seizure outcomes according to the Engel outcome scale were class 1A in three patients, IIA in two patients, IIIA in four patients, and IVA in one patient. Conclusions: AWC can be used for purposes of epilepsy surgery in different situations, either immediately after non-invasive studies or as an additional invasive step after invasive monitoring with either SEEG or SDG. The application of AWC should be individualized according to each patient’s specific characteristics.

## 1. Introduction

Awake craniotomy (AWC) is a method of intraoperative monitoring that is highly valuable for evaluating functions involving the cortical surface and subcortical fibers. The main usage of AWC is currently glioma surgery, attempting maximum tumor removal while preserving brain functions [1]. AWC reportedly increases the removal rate and reduces the complication rate in glioma surgery [2].

In epilepsy surgery, previous studies have reported the use of AWC [3,4,5,6,7]. However, no previous studies have discussed the indications for AWC in epilepsy surgery. This is probably because AWC can be applied in several different clinical situations for epilepsy surgery.

Unlike glioma, epilepsy is a neurophysiological disorder in which surgeons need to integrate observations from multiple imaging modalities, such as electroencephalography (EEG), magnetic resonance imaging (MRI), 18F-fluorodeoxyglucose positron emission tomography (FDG-PET), and magnetoencephalography (MEG). Furthermore, when evaluations for surgical treatment are insufficient after these initial non-invasive evaluations, invasive evaluations using intracranial electrode placement can be performed as the next step. In addition to the evaluation of epileptogenicity, implanted intracranial electrodes enable surgeons to perform functional brain mapping with electrical stimulation. Conventionally, subdural grid electrodes (SDG) have primarily been used, but stereotactic EEG (SEEG) has recently been gaining popularity [8]. The results of intracranial evaluations are considered the most reliable for further resective surgery. Such stepwise diagnostic and treatment flow is a “gold standard” in epilepsy surgery. These steps make the indications and roles of AWC in epilepsy surgery complicated.

We present herein our case series of patients who underwent AWC in epilepsy surgery and propose the indications and roles of AWC in epilepsy surgery.

## 2. Materials and Methods

### 2.1. Patients

Among patients who underwent epilepsy surgery at Osaka Metropolitan University Hospital and Osaka City General Hospital between April 2014 and December 2023, we focused on reviewing patients who underwent AWC. The following patient information was collected: age, sex, etiology, location of epileptogenicity, seizure type, placement of intracranial electrodes, neurological deficits after surgery, requirement for additional surgery, and seizure outcome. Reasons for applying AWC were also reviewed. This study was approved by the institutional review boards at Osaka Metropolitan University Hospital and Osaka City General Hospital.

### 2.2. Diagnostic and Treatment Flow (Figure 1)

**Figure 1:**
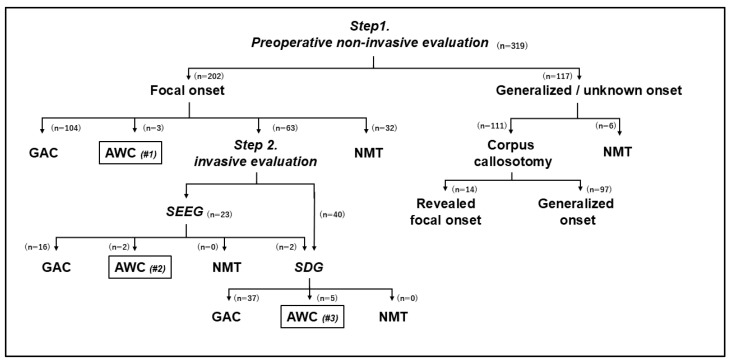
Diagnostic and treatment flow for epilepsy surgery and the number of patients in our series. AWC, awake craniotomy; GAC, general anesthesia craniotomy; NMT, neuromodulation therapy (vagal nerve stimulation); SDG, subdural grid electrode; SEEG, stereotactic electroencephalography.

Epilepsy surgery was indicated when the patient’s seizures had not been controlled even after taking at least two antiseizure medicines for 2 years. Our diagnostic and treatment flow is demonstrated in Figure 1. Step 1 involved non-invasive evaluations, with patients undergoing scalp EEG, MRI, FDG-PET, MEG, neuropsychological tests, and video EEG monitoring to capture seizures. Patients were then divided into two groups depending on these evaluations: those with focal onset epilepsy and those with generalized or unknown-onset epilepsy.

For patients with focal onset epilepsy, when we considered that non-invasive evaluations were sufficient to determine the area of resection, we selected one-stage surgery. The application of craniotomy with general anesthesia (GAC) or AWC was judged considering the age and neuropsychological status of the patient and the location of the area of resection. Indications for AWC in this situation were classified as “AWC#1” in our treatment flow. When we judged that epilepsy could not be cured by any resection surgeries or the patient and their family did not accept a strategy involving craniotomy, vagal nerve stimulation therapy was selected as a neuromodulation therapy (NMT). When we considered that clinical information obtained by non-invasive evaluations was insufficient for determining the area of resection, we proceeded to Step 2, comprising invasive evaluations with placement of intracranial electrodes. Patients with multiple possible epileptogenic lesions, no obvious MRI lesions, and poorly demarcated lesions typically proceeded to this step. Only SDG were available in Japan before 2020, but now we have two methods available using intracranial electrodes: depth electrodes implanted under stereotactic methods (SEEG) and conventional SDG. The type of implanted electrode was determined depending on the clinical information obtained in the non-invasive evaluations of Step 1. However, in cases with the area of resection located around the primary motor area and language-related area, SDG were selected as advantageous for performing cortical brain mapping. Otherwise, SEEG was selected. After evaluations with SEEG, the application of GAC, AWC, or NMT was judged. Indications for AWC in this situation were classified as “AWC#2” in our treatment flow. After SEEG evaluation, if we considered cortical brain mapping as mandatory for determining the area of resection, we then added intracranial evaluation with SDG. After evaluation with SDG, the need to apply GAC, AWC, or NMT was judged. Indications for AWC in this situation were classified as “AWC#3” in our treatment flow.

For generalized or unknown-onset epilepsy, corpus callosotomy (CC) or NMT was applied depending on the results of evaluations and patient preferences, but most patients experiencing drop attacks, tonic seizures, or epileptic spasms were selected to undergo CC. After CC, some patients were revealed as having focal onset epilepsy. For those patients, we performed further evaluations to judge the necessity of intracranial evaluation. Focal resection was then performed.

### 2.3. Evaluations

For the patients who underwent AWC, seizure outcomes were assessed using the Engel outcome scale score as of the most recent follow-up [9].

## 3. Results

### 3.1. Clinical Course of Patients (Figure 1)

Among 319 patients who underwent epilepsy surgery during the study period, 202 patients were diagnosed with focal onset epilepsy, and 117 patients were diagnosed with generalized or unknown-onset epilepsy after Step 1 (non-invasive evaluation). Among the 202 patients who were diagnosed with focal onset epilepsy, 104 patients underwent GAC, 3 patients underwent AWC, 32 patients underwent NMT, and 63 patients proceeded to Step 2 (invasive evaluation). In Step 2 (invasive evaluation), 23 patients underwent SEEG and 40 patients underwent SDG. Among 23 patients who underwent SEEG, 16 patients underwent GAC, 2 patients underwent AWC, and 2 patients underwent additional SDG. The other 3 patients continued medical therapy. Among the 42 patients who underwent SDG, 37 patients underwent GAC, and 5 patients underwent AWC. Among 117 patients diagnosed with generalized or unknown-onset epilepsy, 111 patients underwent corpus callosotomy, and 6 patients underwent NMT. Among 111 patients who underwent corpus callosotomy, 14 patients revealed focal onset epilepsy.

### 3.2. Patients Who Underwent AWC (Table 1)

Ten patients (5 male, 5 female) underwent AWC. The mean age at surgery was 26.2 years (range, 14–41 years). Preoperatively, possible seizure etiologies according to MRI were unknown in 5 patients, low-grade epilepsy-associated tumor (LEAT) in 2 patients, and cavernous malformation, cortical dysplasia, and ulegyria in 1 patient each. The side of the surgery was the left in 7 patients and the right in 3 patients. The location was the frontal lobe in 6 patients, the temporal lobe in 2 patients, and the fronto-parietal lobe and insula in 1 patient each. The seizure type was focal awareness seizure (FAS) with or without focal to bilateral tonic-clonic seizures (FBTCS) in 4 patients and focal impaired awareness seizure (FIAS) with or without FBTCS in 6 patients. Indications for AWC were AWC#1 in 3 patients, meaning that AWC was applied just after Step 1 (non-invasive evaluations). In 2 patients, AWC indications were AWC#2, meaning that AWC was applied after SEEG evaluations. In the remaining 5 patients, AWC indications were AWC#3, meaning that AWC was applied after SDG evaluations. Among these, 2 patients were initially evaluated with SEEG and then with SDG. One patient had slight numbness in the left hand, and one patient showed a slight cognitive decline after surgery, but daily activities were unaffected in either case. Seizure outcomes after AWC according to the Engel outcome scale were class 1A in 3 patients, IIA in 2, IIIA in 4, and IVA in 1. Two patients underwent additional craniotomy because of inadequate seizure control after AWC. They achieved class 1A seizure outcomes after the second surgery. One patient underwent vagal nerve stimulation therapy and achieved relatively better seizure control (class IIIA).

**Table 1:** Characteristics of patients who underwent awake craniotomy for epilepsy surgery.

No.	Age, Sex	Etiology	Side	Location	Seizure Type	Indication of AWC	Neurological Deficit	Seizure Outcome (Engel)	Additional Surgery
1	14, F	Cavernous malformation	R	Frontal	FAS, FBTCS	#1	-	IIIA -> IA	Craniotomy
2	24, M	Foral cortical dysplasia	R	Frontal	FAS, FBTCS	#1	-	IIIA -> IA	Craniotomy
3	41, M	LEAT	L	Insula	FIAS, FBTCS	#1	-	IA	-
4	29, F	Unknown	L	Frontal	FAS, FBTCS	#2	-	IIA	-
5	35, F	Unknown	L	Frontal	FIAS, FBTCS	#2	-	IA	-
6	21, M	Ulegyria	R	Fronto-Parietal	FAS	#3	Left hand numbness	IIA	-
7	24, F	LEAT	L	Temporal	FIAS	#3	-	IVA -> IIIA	VNS
8	25, M	Unknown	L	Frontal	FIAS	#3	-	IIIA	-
9	17, F	Unknown	L	Frontal	FIAS, FBTCS	#3	Cognitive decline	IIIA	-
10	32, M	Unknown	L	Temporal	FIAS, FBTCS	#3	-	IA	-

AWC: awake craniotomy, F: female, FAS: focal awareness seizure, FBTCS: focal to bilateral tonic clonic seizures, FIAS: focal impaired awareness seizures, LEAT: low-grade epilepsy associated tumor, M: male, VNS: vagal nerve stimulation.

### 3.3. Case Illustrations

#### 3.3.1. Patient #1 (AWC#1) (Figure 2)

**Figure 2:**
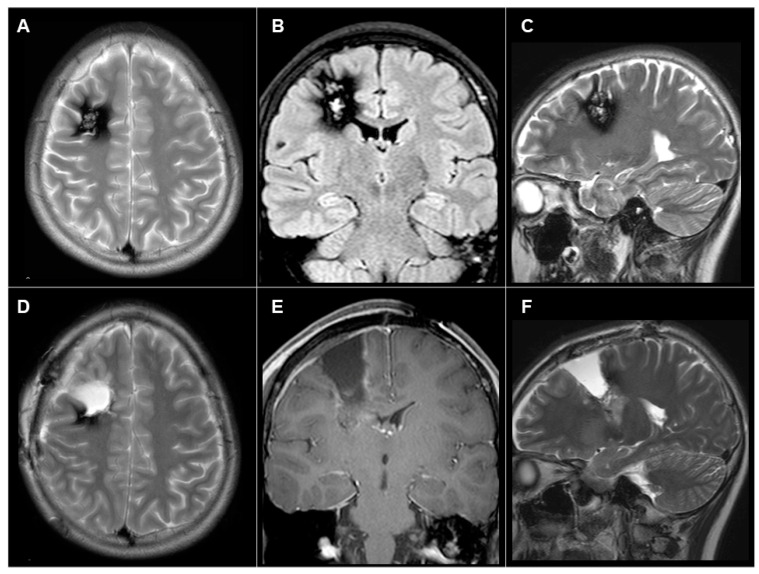
(**A**–**C**) Preoperative axial (**A**), coronal (**B**), and sagittal (**C**) magnetic resonance imaging (MRI) showing the cavernous angioma and surrounding hemosiderin deposit in the right frontal lobe. (**D**–**F**) Postoperative axial (**D**), coronal (**E**), and sagittal (**F**) MRI showing the removal of the cavernous angioma, with some parts of the hemosiderin rim remaining at the posterior edge.

A 14-year-old girl had presented with medically intractable FAS and occasional FBTCS since 10 years old. MRI showed cavernous angioma and surrounding hemosiderin deposits in the right frontal lobe (Figure 2A–C). Cavernoma itself was localized at the middle frontal gyrus, but surrounding hemosiderin deposition extended to the precentral gyrus. EEG showed frequent spikes with maximum amplitude at F4 and C4. To remove the cavernoma and area of hemosiderin deposition, we applied AWC. Positive and negative mapping were performed during AWC to determine the extent of resection. In the deeper part, we also performed subcortical stimulation to identify and avoid damaging the pyramidal tract. After the surgery, the patient did not show any neurological deficits. MRI showed the removal of cavernous angioma, but part of the hemosiderin rim remained at the posterior edge (Figure 2D–F).

The seizure outcome was IIIA according to Engel’s classification. Eight years after the first surgery, we performed an additional craniotomy to remove the residual hemosiderin rim and control the seizures. As of 1.5 years postoperatively, the patient remained seizure-free (Engel class 1A).

#### 3.3.2. Patient #5 (AWC#2) (Figure 3)

**Figure 3:**
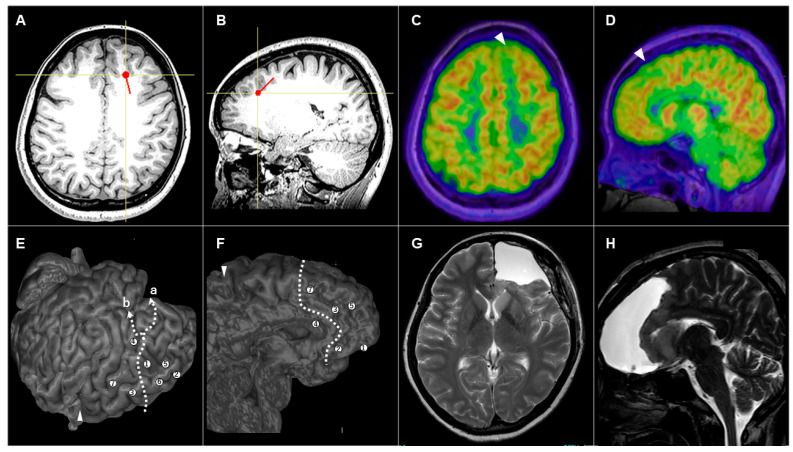
(**A**,**B**) Preoperative axial (**A**) and sagittal (**B**) magnetic resonance imaging (MRI) showing no apparent abnormalities. The red dot and line depict the estimated dipole in magnetoencephalography. (**C**,**D**) Preoperative axial (**C**) and sagittal (**D**) 18F-fluorodeoxyglucose positron emission tomography showing a slightly low accumulation in the left superior frontal gyrus (white arrowhead). (**E**,**F**) Lateral view (**E**) and medial view (**F**) of 3D reconstruction MRI showing the locations of seven SEEG electrodes and resection lines. The posterior margin of resection could not be decided (a or b in (**E**)). (**G**,**H**) Postoperative axial (**G**) and sagittal (**H**) MRI showing the resected area.

A 35-year-old woman had presented with medically intractable FIAS and occasional FBTCS since 17 years old. Interictal EEG showed diffuse spikes bilaterally, with left frontal dominance. Ictal EEG showed spike bursts from the left frontal electrodes. MRI did not show any abnormalities (Figure 3A,B). FDG-PET showed slight low accumulation in the left superior frontal gyrus (Figure 3C,D), and MEG showed dipoles in the same region (Figure 3A,B). We considered that these non-invasive evaluations were insufficient to determine the area to be removed. We therefore proceeded to Step 2 (evaluation with SEEG). We implanted seven electrodes in the left frontal lobe, revealing that seizures originated from the anterior part of the left frontal lobe, including the medial, dorsal, and basal parts. Preoperative electrical stimulation using SEEG electrodes induced anomia at the inferior frontal gyrus (Figure 3E, electrode 4). We set the resection line as shown in Figure 3 (dotted line in Figure 3E,F), but could not decide on the posterior margin of the resection (a or b in Figure 3E). To compensate for the ambiguous mapping results, we applied AWC in focus resection. After the surgery, the patient showed a transient loss of motivation that fully resolved within a week. Postoperative MRI showed the removal of the anterior part of the left frontal lobe (Figure 3G,H). The patient has remained seizure-free for 3 years postoperatively (Engel class 1A).

#### 3.3.3. Patient #10 (AWC#3) (Figure 4)

**Figure 4:**
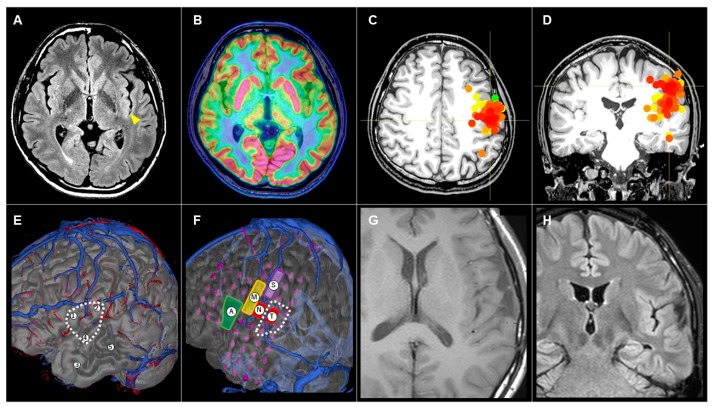
(**A**) Preoperative axial magnetic resonance imaging (MRI) showing a slight signal change in the posterior part of the left insula (yellow arrowhead). (**B**) No areas of low uptake are evident on axial 18F-fluorodeoxyglucose positron emission tomography. (**C**,**D**) Estimated dipoles in magnetoencephalography are demonstrated by red and orange dots on the axial (**C**) and coronal (**D**) images. (**E**) Lateral view of 3D reconstruction MRI. Locations of five SEEG electrodes and the suspected area of epileptogenicity were demonstrated. (**F**) Lateral view of 3D reconstruction MRI. Locations of SDG electrodes, area of induced symptoms, and area of resection, showing hand motor symptoms (M), hand sensory symptoms (S), negative motor symptoms (N), anomia (A), and tongue sensory symptoms (T). (**G**,**H**) Postoperative axial (**G**) and coronal (**H**) MRI showing partial removal of the superior temporal gyrus.

A 33-year-old man had presented with medically intractable FIAS and occasional FBTCS since 26 years old. Interictal EEG showed maximal dull spikes at T3. MRI showed a slight signal change in the posterior part of the left insula (Figure 4A), but this was not a definite finding. FDG-PET (Figure 4B) did not show any areas of low uptake. MEG showed dipoles estimated at the central operculum and insula (Figure 4C,D). We considered these non-invasive evaluations as insufficient to determine the area to be removed. We therefore proceeded to Step 2 (evaluation with SEEG). We implanted five electrodes in the left frontal and temporal lobes (Figure 4E), revealing that seizures originated from the precentral and superior temporal gyrus. The suspected area of epileptogenicity was located in a language-related area (Figure 4E, dotted line). To determine the area of resection, we implanted SDG and evaluated the interictal and ictal EEGs (Figure 4F). We also performed brain mapping with electrical cortical stimulation, which induced motor symptoms, sensory symptoms, negative motor symptoms, and anomia. Those evaluations revealed an overlap between the area to be removed (Figure 4F, dotted line) and the positive areas on electrical stimulation. To determine the area of cortical resection and to evaluate the subcortical language-related tract to be preserved, we applied AWC. Postoperatively, the patient showed no neurological deficits. Postoperative MRI showed partial removal of the superior temporal gyrus (Figure 4G,H). The patient has maintained an Engel class IA seizure-free outcome for 1.5 years.

## 4. Discussion

### 4.1. Summary of the Present Study

Among the 319 patients who underwent epilepsy surgery between April 2014 and December 2023, we applied AWC in 10 patients. We applied AWC under three different conditions (AWC#1 to #3). The percentage of cases undergoing AWC was low (3.1%). Seizure outcome according to the Engel’s outcome scale after AWC was class 1A in 3 cases, IIA in 2, IIIA in 4, and IVA in 1.

### 4.2. Three Different Scenarios for Applying AWC in Epilepsy Surgery

#### 4.2.1. AWC#1

When the epileptogenic lesion is single and located in and around the eloquent cortex and the margin of the lesion is clear on preoperative imaging, AWC can be considered after Step 1 non-invasive evaluations. Cavernous angioma and LEAT are typical etiologies of AWC#1, because these etiologies are visible on MRI and associated with good seizure outcomes with lesionectomy alone [10]. In the surgical removal of cavernoma, cavernoma itself and surrounding hemosiderin deposition should be removed to achieve seizure control. AWC#1 is usually indicated to decide the margin of corticotomy and confirm white matter tracts running in deeper parts.

#### 4.2.2. AWC#2

When the area of planned resection overlaps the eloquent cortex as estimated from SEEG, AWC can be considered. Extraoperative electrical stimulation mapping with SEEG electrodes can provide information about cortical and subcortical functions but is mostly insufficient to decide the margins of resection. To compensate, information obtained from stimulation mapping during AWC is a useful option. AWC#2 is usually indicated for non-lesional or ill-demarcated lesions to define the border of resection.

#### 4.2.3. AWC#3

Even after SDG evaluation, which offers mapping results from electrical stimulation, AWC can be considered. AWC#3 is indicated when the planned resection area is within the eloquent cortex, and meticulous differentiation between the area to be removed and the eloquent cortex is needed to preserve brain functions. AWC#3 is also indicated when U-fibers connecting to the adjacent cortex should be preserved.

### 4.3. Future Directions of AWC in Epilepsy Surgery

The proportions of usage of SDG and SEEG have been changing since the introduction of SEEG [11,12,13,14]. In our institutions, after the Japanese government approved SEEG in 2021, most patients (68%, 17/25) underwent SEEG as the initial part of Step 2 (invasive evaluation with intracranial electrodes). SEEG has the advantage of being less invasive but also has the disadvantage of providing lower spatial resolution in brain mapping. AWC can compensate for insufficient information from brain mapping by SEEG. In the present study, the percentage of patients undergoing AWC in epilepsy surgery was low (3.1%), but we assume that the number of patients undergoing AWC will increase along with the increasing number of patients undergoing SEEG. AWC is generally applied to patients between 15 and 65 years old [2], and the proportion of young patients is much higher in epilepsy surgery than in glioma. The relatively young cohort thus represents a key limitation to the generalizability of these findings regarding AWC in epilepsy surgery.

### 4.4. Limitations

The present study was a single-center retrospective study demonstrating three different scenarios in which AWC can be considered for epilepsy surgery. However, comparing seizure outcomes between these scenarios is difficult because of the small number of patients. Future studies with larger patient cohorts are needed to validate the efficacy of each scenario for AWC in epilepsy surgery.

## 5. Conclusions

Among 319 patients who underwent epilepsy surgery, we demonstrated 10 who had AWC and proposed three different scenarios for applying AWC in epilepsy surgery. Surgeons should understand the purpose of AWC in each patient and decide on a surgical strategy to achieve good seizure control and functional preservation.

## Data Availability

No new data were created or analyzed in this study. The data presented in this study are available on request from the corresponding author due to privacy reasons.
